# Network mechanisms of grid cells

**DOI:** 10.1098/rstb.2012.0511

**Published:** 2014-02-05

**Authors:** Edvard I. Moser, May-Britt Moser, Yasser Roudi

**Affiliations:** Centre for Neural Computation, Kavli Institute for Systems Neuroscience, Norwegian University of Science and Technology, MTFS, Olav Kyrres gate 9, NTNU, 7489 Trondheim, Norway

**Keywords:** hippocampus, entorhinal cortex, grid cells, cortex, spatial representation, attractor network

## Abstract

One of the major breakthroughs in neuroscience is the emerging understanding of how signals from the external environment are extracted and represented in the primary sensory cortices of the mammalian brain. The operational principles of the rest of the cortex, however, have essentially remained in the dark. The discovery of grid cells, and their functional organization, opens the door to some of the first insights into the workings of the association cortices, at a stage of neural processing where firing properties are shaped not primarily by the nature of incoming sensory signals but rather by internal self-organizing principles. Grid cells are place-modulated neurons whose firing locations define a periodic triangular array overlaid on the entire space available to a moving animal. The unclouded firing pattern of these cells is rare within the association cortices. In this paper, we shall review recent advances in our understanding of the mechanisms of grid-cell formation which suggest that the pattern originates by competitive network interactions, and we shall relate these ideas to new insights regarding the organization of grid cells into functionally segregated modules.

## 1. Introduction

Grid cells are place-modulated neurons with discrete firing fields arranged in a periodic hexagonal lattice [1–3]. The unit of the tessellating pattern is an equilateral triangle, such that the fields of the grid pattern are spaced at equal distances and at angles of 60°, independently of the animal's speed and running direction at each location in the environment [1]. Grid cells vary along three key parameters: the phase of the grid (the *x*–*y* coordinates of the grid vertices), the spacing of the grid fields (grid frequency), and the orientation of the grid axes [1]. Grid cells are particularly abundant in layer II of the medial entorhinal cortex (MEC) but also exist in somewhat smaller quantities in deeper MEC layers and in the adjacent pre- and parasubiculum [4,5].

Grid cells can be distinguished from hippocampal place cells, which also fire at specific locations [2,6]. Place cells differ from grid cells not only in their lack of periodic firing fields but also in the apparently random relationship between the firing fields of different neurons. In the hippocampus, different combinations of place cells are active in different environments and to the extent that there is overlap between any active cells, their firing locations tend to be different [7,8]. By contrast, grid cells are always active and clusters of grid cells generally maintain their relative firing locations across environments [9]. Phase and orientation differences between two neighbouring grid cells tend to carry over from one environment to another, such that if the grid fields of two cells are out of phase in one room, they will be out of phase in the other room too. A similarly rigid structure has been observed in other co-localized MEC cell types, such as head direction cells and border cells, whose firing fields retain relative orientations from one environment to the other [10,11]. Absolute parameter values for grid cells, head direction cells and border cells are set uniquely for each environment, probably by associative plasticity, but within local cell clusters, relative differences between cells tend to be preserved. The rigidity of the MEC map provides the brain with a representation of distances, directions and geometric relationships that can be read out similarly in all environments.

The aim of this paper is to review advances in our understanding of the neural network operations underlying the grid pattern. In the first part, we shall demonstrate how converging evidence points to a mechanism for grid patterns where grids emerge through competitive inhibitory interactions between grid cells with similar scale properties. In the second part, we shall review recent work suggesting that the MEC consists of multiple grid networks or maps operating on a partly independent basis. We shall argue that independently operating grid modules may form the basis for high-capacity memory downstream in the hippocampus.

## 2. Mechanisms of grid cells

A number of models have been proposed to explain the formation of grid patterns in MEC cells. In nearly all of these models, periodic firing locations are determined by path integration of velocity-modulated input signals, with other sensory inputs contributing only to setting the initial phase and orientation of the grid [2,3]. However, this does not, in principle, rule out the possibility that grid patterns could be formed from spatial inputs only [12].

In one class of models, referred to as the oscillatory interference model, the spatially periodic firing of a grid cell emerges within the cell as an interference pattern between a relatively constant theta oscillation and other internal or external velocity-controlled theta oscillators [13–20]. The frequency of these latter oscillators is taken to be modulated by the instantaneous running speed of the animal along a preferred direction. The interference of each such oscillator with the reference membrane oscillation leads to the formation of bands of activity with the same orientation as the preferred orientation of the corresponding velocity-modulated oscillator. Consequently, if the orientations of the velocity-controlled oscillators are separated by 60°, one expects to observe a hexagonal periodic spatial activity.

Oscillatory interference-based models have successfully explained some of the temporal firing properties of grid cells, such as theta phase precession [21–23], but the models are facing serious challenges as a candidate mechanism for spatial periodicity. A major problem is that the 60° separation has to be put into the model by hand, i.e. the models explain the regularity in the organization of grid cells’ spatial firing fields by assuming a similar regularity in the preferred direction of the oscillators. Although it has been argued that self-organizing mechanisms can yield the 60° separation [13,15], no implementation of such a mechanism is yet offered. Given that a separation of 60° is the defining feature of a hexagonal grid pattern, any model should ultimately aim at explaining how it emerges from single cell or network mechanisms without assuming it in the first place.

In addition, when it comes to experimental testing of whether the ingredients of the model exist, studies have failed to verify two of its key assumptions. One of these is that grid cells require theta oscillations. Grid cells have now been demonstrated in Egyptian fruit bats, which lack the persistent theta oscillations characteristic of hippocampal and parahippocampal regions in rodents [24]. In bats that crawled on a surface, the spatial periodicity of the grid cells persisted during periods when no theta waves or theta modulation could be identified in the recorded activity. Grid cells have also been observed in monkeys, which also have only intermittent theta oscillations [25]. As in bats, grid cells were equally periodic in the presence and the absence of theta bouts. A second set of studies has tested the prediction that, in the rodent brain, where theta oscillations are indeed present, grid fields should coincide with large-amplitude theta-interference waves in the cell's membrane potential. These studies performed whole-cell recordings in entorhinal stellate cells of head-fixed mice navigating in a virtual-reality environment [22,23]. Contrary to predictions of the oscillatory interference models, the periodicity of the firing locations was only minimally associated with changes in the amplitude of theta oscillations in the cell's membrane potential, although the spike timing reflected the theta rhythmicity. Grid fields were instead accompanied by ramps of depolarization lasting as long as it took the animal to pass through the firing field. Taken together with the theoretical limitations of the interference models, these experimental observations in our view invalidate oscillatory interference as a mechanism for spatial periodicity in grid cells (but see [26,27] for an alternative position).

An alternative class of models suggests that grid patterns are formed in local circuits with attractor properties. Common to this class of models is the suggestion that hexagonally patterned activity emerges as a consequence of interactions between recurrently connected neurons, and that neural activity moves across the connection matrix in accordance with the animal's movements through the environment [28–30]. An important feature of these models is that the hexagonal regularity emerges from the network without a need for putting it in at any level.

**Figure 1.**
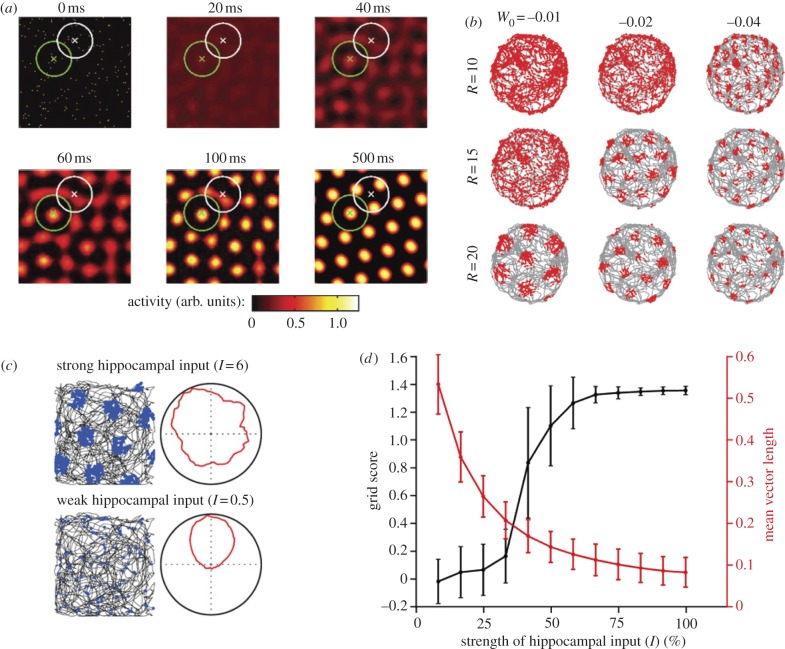
Inhibition-based attractor network model for grid cells. (*a*) A hexagonal grid pattern forms spontaneously (here over a period of 500 ms) on a two-dimensional neuronal lattice consisting of stellate cells that have all-or-none inhibitory connections with each other. Neurons are arranged on the lattice according to their spatial phases. Activity is colour-coded, as indicated by the scale bar at the bottom. Connection radii *R* of two example neurons are shown as white and green circles (diameter 2*R*). Note lower activity where the circles overlap (at 500 ms). (*b*) Simulated single neuron activity (red dots) over 10 min of foraging in a 1.8 m diameter circular arena. *W*_0_ is the strength of the inhibitory connectivity of the network; *R* is the radius. Note that *W*_0_ and *R* control the size of the grid fields and their spacing. (*c*) Effect of excitatory drive from the hippocampus. Spike distribution plots (as in *b*) and directional tuning curves (firing rate as a function of direction) for two example cells in the presence of strong hippocampal output (top) and weak hippocampal output (bottom). (*d*) Grid scores (sixfold rotational symmetry) and mean vector length (directional tuning) as a function of the strength of external input (means ± s.e.m.). With large hippocampal inputs, high grid scores are obtained, as in the top image in (*c*). When the external input is decreased below a critical amount, as in the bottom image of (*c*), the activity on the neuronal sheet gets easily distorted and hexagonal structure is not detectable over time. At the same time, the head-directional input becomes the dominant source of input and the neurons show high directional tuning. (*a*,*b*) Adapted from [32]; (*c*,*d*) from [33].

In the earliest attractor models for grid cells, periodic firing fields were proposed to result from Mexican-hat connectivity, i.e. strong excitatory connections between cells with similar grid phases and progressively weaker connections between cells with increasingly different phases [28,29]. A surrounding field of global inhibition prevented the local excitation from spreading. However, the validity of these models relies on the assumption that the entorhinal network actually has the recurrent connectivity required for spontaneous formation of hexagonally patterned firing fields. Early experimental studies indicated an almost complete lack of excitatory connections between stellate cells in MEC layer II [31], where the number of grid cells is largest [4,5]. This observation has been recently verified in whole-cell patch recordings from more than 600 pairs of stellate cells in MEC layer II. By evoking action potentials in one or several simultaneously patched stellate cells and recording postsynaptic potentials in others, Couey *et al*. [32] demonstrated a bimodal connectivity pattern where all functional connections were inhibitory. Simulations showed that competitive inhibitory interactions, with a constant magnitude and a fixed radius, are sufficient for neural network activity to self-organize into a stable hexagonal grid pattern (figure 1*a,b*). On a sheet of neurons arranged according to spatial phase, activity emerged at maximally spaced positions, i.e. at the vertices of a hexagonal grid. The activity could then be translated by the same mechanism as in the previous attractor models. These findings are in agreement with independent results from another study [34], which also found that connections between stellate cells are almost exclusively inhibitory and which showed, in a two-population (excitatory–inhibitory) attractor model of spiking neurons, that grid activity and gamma oscillations can emerge in the same network.

The fact that a purely inhibitory network can generate grid cells has already been noted by Burak & Fiete [30]; however, the connectivity of their model still follows a Mexican-hat shape in the sense that cells with similar phases inhibit each other less and inhibition then increases as phase differences increase, after which the inhibition again decays to zero. The all-or-none connectivity in Couey *et al*. [32] could be seen as a special case of the Burak-and-Fiete weight matrix in which the amplitude of the positive Gaussian is zero (*a* = 0 in their eqn 3) and the negative Gaussian has steep flanks. In any case, the hexagonal pattern emerges as an optimally packed arrangement of inhibition circles surrounding activity bumps. Although no fundamental differences exist between the two models in terms of the mechanism that they employ for the generation of grid cells, the attractive feature of the connectivity used by Couey *et al*. is that it would be easier to generate such connectivity developmentally. Taken together, these sets of studies suggest that connections do not have to be Mexican-hat type and that all-or-none inhibitory connectivity is sufficient for grid formation.

A potential problem is that a purely inhibitory network depends on tonic external excitatory drive for keeping cells sufficiently near firing threshold to fire. Such excitatory input can come from a variety of sources, one of which could be the hippocampus, which, via the deep MEC layers, has strong projections back to the superficial layers of the MEC [35,36]. Recent work has shown that entorhinal grids disappear after inactivation of the hippocampus [33]. The loss of grid patterns is contingent on a drop in the firing rate of the grid cells and coincides with the appearance of directional tuning in grid cells that were initially non-directional. These effects could all be replicated by removing excitatory drive in the inhibitory attractor network model (figure 1*c,d*). In the absence of intrinsic inhibitory influences, the former grid cells settled in a state where they responded primarily to external directional inputs. The excitatory drive may also come from other sources such as the medial septum, whose activity is necessary for maintenance of grid activity in the MEC [37,38]. A similar effect of removing excitatory input might not be observed in networks with Mexican-hat connectivity, where excitation might be provided within the network itself [28,29].

It is worth noting that if the periodic firing of the grid cells gets disrupted by blockade of external excitatory drive, it may still be possible, under certain conditions, to observe short periods of grid-like activity on the neuronal sheet. In particular, grid patterns should be detectable when the animal moves in a constant direction. For cells whose preferred direction is along the animal's movement, the head-directional input may then momentarily compensate for the missing external drive. Because the activated population changes as the animal changes direction, a grid-like firing pattern would not be apparent over time (supplementary movie in [33]). However, if many cells are recorded simultaneously and the cells are arranged according to their phases, a hexagonal grid pattern should be detectable momentarily provided that the level of noise or other inputs is not too strong.

The main problem with the attractor framework in our view is that of the sensitivity to noise in recurrent connectivity. Static noise in the recurrent connectivity is known to result in unwanted drift in the activity of continuous attractors [39]. Such drift would destroy the grid pattern, although different connectivity patterns (e.g. Mexican-hat versus all-or-none) may have different levels of noise tolerance. One way to remedy the drift involves the use of gain modulation [40] or synaptic facilitation [41]. Synaptic facilitation has been shown to alleviate drift in one-dimensional ring attractor models of working memory [41]. Such ring attractors may in turn self-organize into two-dimensional grid-cell attractor networks. Grossberg and colleagues [42] have developed a model in which at a first layer, using one-dimensional ring attractors, cells with stripe-like spatial selectivity are generated. These ring attractors interact with each other through a self-organizing mechanism and a plasticity rule to achieve 60° separation in the stripe directions. When the activity of the cells in the ring attractors is projected to a postsynaptic cell, the cell will respond with regular firing fields organized as a hexagonal lattice. One would still like to know how the connectivity required for the one-dimensional ring attractor is developed, but answering this question is simpler than that of a two-dimensional continuous attractor network. A full model combining ring attractors with imperfect connectivity and synaptic facilitation or gain modulation with a self-organizing mechanism is yet to be worked out but given this previous work it may be a straightforward extension.

The progress recently made in understanding the circuitry of the grid cell network has paved the way for answering an important question that so far has been largely neglected, which is how grid cells are formed during maturation of the nervous system. Since nearby grid cells do not seem to have nearby phases, a connectivity formed topographically during development is not likely to provide the right answer. At the moment, the only explicit attempt to offer a different mechanism for the development of grid cells is the work by Kropff & Treves [12], where the authors show that spatially modulated signals from plastic synapses, in conjunction with adaptation, can generate individual grid cells. These grid cells have random orientations, contrary to experimental observations. However, if combined with recurrent excitatory connectivity, the grid pattern of individual cells can become oriented to the same direction [12,43]. A crucial question at this stage is whether this adaptation model can operate within the inhibitory network of the MEC and, more importantly, if connectivity similar to the experimentally observed pattern can be learnt in an unstructured network endowed with Hebbian plasticity on recurrent connections and subject to mildly space-modulated external input.

## 3. Modular organization of grid cells

Early studies of grid cells suggested that the grid network operates as a single unified network in which cells responded coherently to changes in the environment [1,9]. The existence of a single continuous network poses challenges for all attractor network models, however, because the proposed correspondence between velocity of movement in the environment and displacement in the neural sheet can probably only be maintained if the grid network has a common grid scale and grid orientation. Grid spacing and grid orientation vary widely, with spacing ranging from 20 to 30 cm to several metres [44] and orientation sometimes differing by 30° [45,46]. Given such large variations, it is hard to imagine how a single network, even with continuously varying properties, could sustain all different scales and orientations. Discretization to multiple networks, each with relatively stable anatomy (e.g. same connectivity pattern) and physiology (e.g. same velocity couplings) within each module, but not between modules, would be a simple way to deal with this problem.

**Figure 2.**
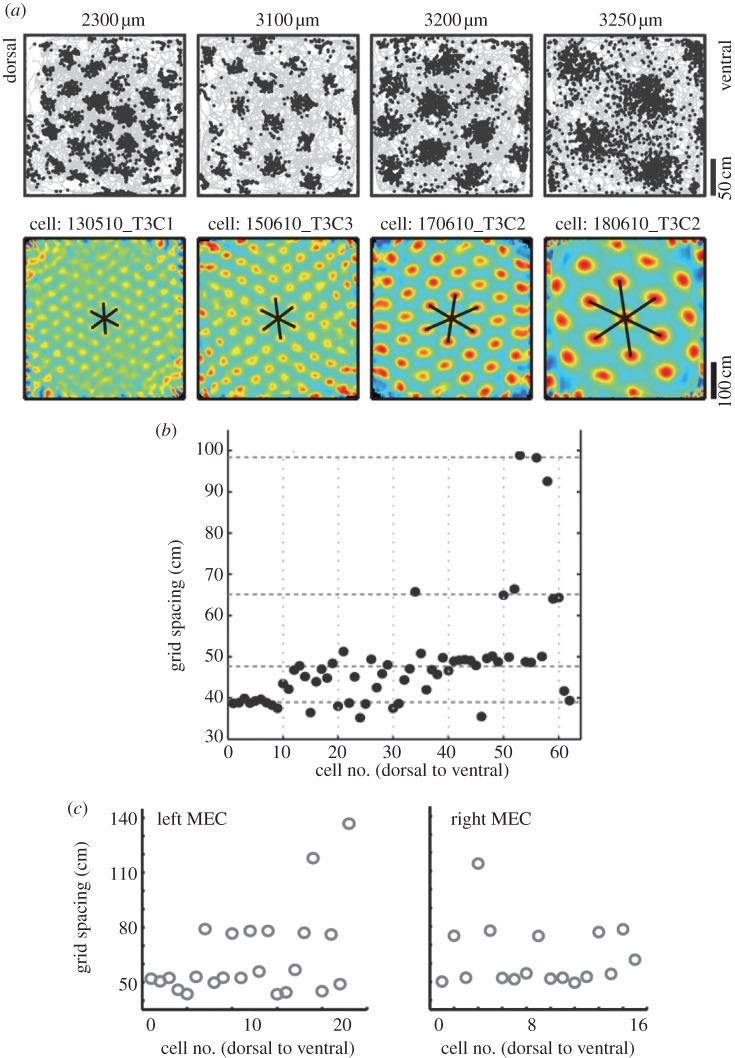
Modular organization of the grid map. (*a*) Examples of grid cells belonging to modules with different grid spacing. Left to right: Spike maps (top row) and colour-coded autocorrelation maps (bottom row) for four example cells. Trajectory of the rat is shown in grey, with all of the cell's spike locations superimposed in black. Colour scale for the autocorrelation maps is from blue (*r* =−1) to red (*r* = 1). Recording depth along the dorsoventral axis of MEC is indicated at the top. (*b*) Step-like organization of grid scale. Grid spacing is shown as a function of position along the recording track in MEC, with cells (dots) rank-ordered from dorsal to ventral. Note that grid spacing clusters into four different values in this recording. Horizontal lines indicate mean values for *k*-means-identified clusters. (*c*) Step-like changes in grid spacing in grid cells recorded simultaneously in left and right MEC. Cells are numbered according to position along the dorsoventral MEC axis. Note discrete nature of the distributions as well as identical values for grid spacing in the two locations. (Adapted from [46].)

Until recently, low numbers of simultaneously recorded grid cells have prevented a clear answer to the question of whether the grid-cell system operates as one integrated network or as a collection of discretized modules. We have recently been able to increase the number of grid cells recorded in a single animal by an order of magnitude, from around 10–20 to almost 200 [46]. This allowed us to determine whether grid cells recorded at high density across a wide region of MEC cluster into modules with distinct scale and orientation, as required for accurate translation of activity in the network, or if grid cells were organized with smooth gradients, as would be possible if the grid pattern was caused entirely by intracellular mechanisms. Nearly 1000 cells were recorded from 15 rats, using tetrodes that followed layers II and III tangentially or accessed a large number of positions simultaneously. Grid cells were recorded over a stretch of almost 2 mm along the dorsoventral axis of the MEC, covering more than one-third of the length of MEC in this direction, and over a distance of 1 mm in the mediolateral direction, corresponding to almost 50% of the mediolateral extent along the dorsal border of MEC. Discrete steps in grid scale were observed in every single animal (figure 2). Clusters with different grid scales also tended to have different grid orientations. The clusters spanned layers, such that deep and superficial cells generally had similar scale and orientation properties. The modular nature of the grid map confirms a key prediction of the attractor models.

How many grid modules are there? So far, large numbers of grid cells have only been recorded from the dorsomedial one-third of the MEC. Yet, by extrapolation, the data suggest that the total number of grid modules is small, probably less than 10. The existing data generally clustered into three to four values with large gaps between each step. In one animal, a fifth module was probably present but the firing fields of these cells were too large to determine conclusively whether they were part of a grid pattern. Modules with small values for grid spacing predominated at the dorsal-most recording positions. Then as the recording electrodes were moved in the ventral direction, successively larger grids were recruited. No gradients were observed in the mediolateral direction, suggesting that the modules are organized as horizontal bands parallel to the dorsal border of the MEC. There was considerable anatomical overlap between the clusters, particularly at more ventral locations, suggesting that grid modules are entangled, quite unlike the graded topography of many maps for continuous functions in the primary sensory cortices [47,48].

The scale relationship of successive grid modules shows some interesting features that may be relevant to how effectively the spatial environment is represented. On average, grid spacing increased from one module to the next by a factor of 1.42, or the square root of 2. The geometric progression defined by this scale relationship enables the inner ring of the grid hexagon to exactly double from one module to the next. Interestingly, theoretical analysis suggests that in order to estimate the position from the response of grid cells with high resolution, such a modular organization with geometric scaling of grid spacing is required [49].

How does grid modularity come about? In the attractor networks, there are two factors that control field size and grid spacing. The first one is the strength of the velocity-dependent head-directional signal: increasing this signal results in faster translation of the grid pattern on the sheet as the animal moves and thus makes the spacing between single cell grid fields smaller. If this is how grid scales become different across modules, the MEC may have multiple path-integrating attractor networks with similar recurrent connectivity patterns but each operating with a different amplification of the incoming speed signal. Another way to change grid spacing and field size is to alter the strength or the extent of recurrent connections. As shown in figure 1, changing the width or the radius of the inhibitory connectivity changes the spacing between the fields in stationary grid pattern. Consequently, with this mechanism, modularity suggests that the grid network comprises different sub-networks with dissimilar connectivity patterns. Detailed anatomical studies as well as experiments studying how grid cells within the same module and grid cells in different modules react to changes in environment will be necessary to distinguish between these different possibilities.

The existing data suggest that grid modules can operate independently. Changes in grid scale coincide not only with changes in grid orientation but also with differences in elliptic distortions of the grid, as well as variations in theta-frequency modulation of the grid cells [46]. Moreover, when the recording environment is compressed by transforming the familiar square box into a rectangle, cells in different modules may respond independently. Following compression of the recording box, grid cells in the smallest module showed no reorganization, whereas all cells in the larger modules rescaled completely, maintaining the original number of grid fields in the compressed direction with smaller inter-field distances [46]. These data provide proof-of-principle evidence for the idea that grid modules can operate independently in response to external information.

The organization of grid cells into independent modules may have consequences not only for representation of the animal's current location in the MEC but also for the network's ability to store memory based on grid maps. Hippocampal place cells are likely to receive convergent input from multiple grid modules. If these converging modules respond independently during transitions to a different environment, or when an individual environment is sufficiently altered, the altered co-activity of entorhinal grid cells will likely activate a new subset of place cells at each location in the new environment [9,46,50]. Every relative shift in phase, orientation or scale of two or more grid modules might lead to a complete replacement of the active hippocampal place-cell population. Independent displacements among grid modules might thus give rise to a large number of different coactivity patterns in the hippocampus. Computational simulations have suggested that the number of modules required to achieve such pattern expansions is very small, not very different from the number of modules observed in existing data [51]. The formation of large numbers of coactivity patterns in the hippocampus, as a result of independently operating grid modules, may have been the mechanism that during evolution allowed the hippocampus, originally evolved to map the spatial environment, to take on an increasingly important role in episodic memory formation, where storage of vast numbers of activity patterns is fundamental [50].

## Funding statement

The work was supported by the Kavli Foundation, a Centre of Excellence grant from the Research Council of Norway, an Advanced Investigator Grant to E.I.M. from the European Research Council (‘CIRCUIT’, grant agreement 232608), and a grant from the European Commission's 7th Framework 2011 ICT Programme for Future Emerging Technologies (‘GRIDMAP’, grant agreement 600725).
